# I want to talk about climate change, but I wish I didn’t have to: A descriptive qualitative study of an intervention combining creative arts and philosophical inquiry to help elementary school students cope with climate change emotions

**DOI:** 10.1186/s40359-025-03589-w

**Published:** 2025-12-08

**Authors:** Terra Léger-Goodes, Catherine M. Herba, Jasmine Piché, Jonathan Smith, Marc-André Éthier, David Lefrançois, Catherine Malboeuf-Hurtubise

**Affiliations:** 1 Department of Psychology, Université du Québec à Montréal (UQAM), Montreal, QC Canada; 2 Azrieli Research Center of the CHU Sainte-Justine, Montreal, QC Canada; 3 Department of Psychology, Bishop’s University, Sherbrooke, QC Canada; 4 Department of Preschool and Primary Education, Université de Sherbrooke, Sherbrooke, QC Canada; 5 Research Centre of the CHU Sherbrooke, Sherbrooke, QC Canada; 6 Faculty of Science of Education, Université de Montréal, Montreal, QC Canada; 7 Department of Sciences of Education, Université du Québec en Outaouais, Saint-Jérôme, QC Canada; 8 School of Psychology, Université Laval, Québec, Qc Canada

**Keywords:** Climate change emotions, Eco-anxiety, Children’s mental health, Art-based intervention, Philosophy inquiry, Existential psychology, Positive psychology

## Abstract

**Background:**

Children are increasingly exposed to climate change impacts through school curricula, media, and in their environments, leading to various emotional responses, including sadness, anger, and fear. While such emotional reactions to truly distressing situations are normal, they can disrupt daily functioning. The implementation of creative arts and philosophical inquiry in elementary schools could foster adaptive coping through meaning-making activities, namely, by providing spaces for introspection, emotional expression, and exploration of existential questions. This study aimed to document the social validity of an intervention combining creative arts and philosophical inquiry and to examine its acceptability, the perceived goals, and perceived benefits for children’s mental health and ability to cope with climate change.

**Methods:**

Using a descriptive qualitative design, this study captured students’ and teachers’ perspectives through semi-structured interviews, group discussions, and observations. Thematic analysis was employed to evaluate the intervention’s acceptability and perceived mental health effects.

**Results:**

Two main themes emerged, supporting the intervention’s social validity. First, the participants emphasized the importance of climate change discussions in the classroom, with both students and teachers reporting appreciation of the creative arts and philosophical inquiry components. There were no negative impacts reported by either children or teachers. Second, with respect to intervention effects, children reported various emotional responses, with some noting that while difficult emotions remained, they felt better equipped to cope with them. The intervention appeared to support students’ psychological needs for autonomy, competence, and affiliation.

**Conclusions:**

The creative arts and philosophical inquiry intervention demonstrated strong social validity. The children’s feedback highlighted the need for safe and brave spaces to explore issues related to climate change and to express difficult emotions such as despair. These findings highlight the importance of implementing such programs in schools and providing potential tools for educators. Since children will bear the impacts of climate change in the years to come, it is crucial to foster adequate coping mechanisms and create spaces for discussion, connection, and emotional expression.

**Trial registration:**

This study has been retrospectively registered on January 17th, 2025. Trial registration number: NCT06781788.

**Supplementary Information:**

The online version contains supplementary material available at 10.1186/s40359-025-03589-w.

## Background

Climate change emotions among children represent an emerging area of research, reflecting growing awareness of global environmental changes [1–5]. A comprehensive cross-sectional survey among 10 000 youths between the ages of 16 and 25 years across ten countries indicated that 27% were extremely worried about climate change, 32% were very worried, and 45% reported that their worry affected their daily functioning [6]. This concern has often been referred to as eco-anxiety, which can be defined as a “chronic fear of environmental doom” [7]. However, the emotional experience of climate change can include a broad range of emotions beyond fear and anxiety, including grief, sadness and hope [8, 9]. Hence, the term “climate change emotions” can be used to refer to the spectrum of emotions that may arise from the awareness of climate change.

Most research has documented climate change emotions in adults (e.g., [10, 11]), but evidence also suggests that children experience these same emotions [3, 4]. For example, a qualitative study in Canada revealed that children reported feeling sad for animals and humans who experienced the direct consequences of climate change, feeling angry toward older generations and polluters, scared and apprehensive for their future, and grateful that they had not yet experienced too many direct environmental consequences [12]. Furthermore, a narrative review of the experience of eco-anxiety in youth highlighted that narratives of doom, general pessimism, and cynicism about the future are common across the literature [13]. These emotional experiences are not viewed as pathological but rather are understood as dynamic, context-dependent responses that exist on a complex psychological continuum [14–18]. In some instances, these feelings serve as adaptive mechanisms, motivating meaningful pro-environmental engagement and action [19–21]. Conversely, for some individuals, these emotions can become overwhelming, potentially leading to psychological distress [17, 22]. While existing research has begun to explore the clinical implications of eco-anxiety, particularly in adult populations [23], a better understanding of climate change emotions and how we can help children navigate these challenging times are needed [13].

### Coping with climate change emotions

Coping has been defined as a process of adapting to the challenges, stresses, and demands of life [24]. Different coping strategies can be used to address difficult circumstances on the basis of individual characteristics, past experiences, and the nature of the stress [25]. Adaptive coping can contribute to the maintenance of good mental health and reduce the physical and mental impacts of stress [26]. In the process of adapting to the reality of climate change, Pihkala [9] identified three essential dimensions: action, grieving, and distancing. Hence, children should be offered opportunities to act in their community in order to feel that they have power and can contribute to solving the problem. They should also be allowed to grieve, which includes identifying, engaging with, and sharing all climate change emotions in a safe space where they feel these emotions are validated. Finally, children should also learn to distance themselves from the problem of climate change to take care of themselves and to sometimes avoid climate change related information, when appropriate.

Ojala highlighted that meaning-making, or making sense of the situation, emerges as a crucial adaptive strategy for navigating the complex emotional landscape of climate change [27, 28]. This approach involves reframing environmental challenges through a nuanced lens that acknowledges both negative and positive emotions, including hope, gratitude, love, and contentment. Notably, meaning making involves welcoming and making space for both positive and negative emotions [29]. In this vein, meaning can be fostered by identifying and naming existential challenges posed by climate change and its associated emotions while reframing the narrative to gain a greater sense of internal coherence or alignment with emotions, thoughts, and behaviors [30].

### Interventions to support coping with climate change

Reviews of initial interventions to foster adaptive coping in adults in the context of climate change emphasized the potential of social connection, emotional expression, emotional validation, and the creation of meaning [13, 31, 32]. However, these reviews also highlight the lack of studies evaluating the effects of such interventions. With respect to children, the existing literature predominantly focuses on climate change education (e.g., [33]), with limited tools available to address climate change emotions in the classroom [34, 35]. Photovoice emerges as an artistic approach that has been used to facilitate children’s emotional expression through photography, allowing them to share their story and, for others looking at it, to create meaning from others’ experiences [36, 37]. In the field of clinical psychology, photovoice is a therapeutic approach aimed at promoting citizen action, civic responsibility, global social change and artistic expression [38]. With children, using photovoice to discuss the difficult topic of climate change also allows for fun and enjoyment while remaining actively engaged with the issue [39]. Hence, this approach could offer children the opportunity to explore climate change emotions in a group setting such as the classroom through photography while supporting dialogue, meaning making, and action.

Other creative approaches can also foster emotional awareness and expression [40]. For children, creating visual art supports emotional expression and can be more accessible than verbal communication to express their emotions [41–43]. Using creative arts allows individuals to reframe their experiences and create a sense of coherence in their lives [44]. Creating art also promotes a sense of control over emotions [45]. Using creative arts to express climate change emotions could lead to a sense of control and release of difficult emotions [46], yet no study, to our knowledge, has evaluated this phenomenon.

While emotional expression through art can prove soothing, existential questions may persist within the context of climate change, since the problem cannot be solved [46, 47]. Approaches rooted in philosophical inquiry could address this gap. Such interventions use existential questions to explore values, reflect on a common problem, communicate ideas, co-construct meaning, and address certain ambiguities [48, 49]. These group discussions guided by a given theme (e.g., our responsibility toward the planet) allow the exploration of existential issues and questions. Preliminary research suggests that such approaches can foster hope and support emotional processing [50, 51]. In turn, this may promote space for questions and inquiry around the existential components of climate change and help children cope by creating meaning. While philosophical inquiry can raise certain difficult and destabilizing questions, addressing this existential anxiety rather than ignoring it could support adaptive forms of coping [52]. Combining creative arts and philosophical inquiry could be an ideal combination, as they allow individuals to explore their cognitive and emotional responses to climate change, both of which are essential for adaptive coping [20, 53]. Adaptive coping can be further strengthened when individuals can engage with the issue of climate change and align their choices with their needs [54], which is linked to self-determination and discussed in the next section.

### Supporting the satisfaction of basic psychological needs in the context of climate change

The present intervention is rooted in self-determination theory, which highlights the importance of three basic psychological needs: the needs of autonomy, competence, and affiliation. The satisfaction of these psychological needs contributes to the general well-being, motivation, and optimal functioning of individuals of all ages [55]. The need for autonomy refers to the feeling of being in control of one’s own choices and actions so that they correspond to personal values and interests [55]. By fostering autonomy, young people can learn to identify the values and emotions that guide their choices, giving them a sense of power over their actions in the context of climate change. The need for competence refers to the need to feel effective and able to have an impact on one’s interactions with the environment [55]. Encouraging children to take on challenges, giving them constructive feedback and recognizing their successes all contribute to the development of a sense of competence, which has a positive impact on their well-being [56]. Finally, the need for affiliation refers to the need for connection and belonging to others (i.e., to love and be loved). Social interactions and positive social environments are recognized as significant factors contributing to children’s well-being [57, 58]. Self-determination theory was used as a theoretical frame and guided the development of the intervention to ensure that the elements included would support the basic psychological needs of children throughout the workshops. For example, elements of choice were built in the intervention, including choice in materials and topics for their creations to support autonomy; challenge level was adapted to different children with mediums accommodating different skill levels to support competence; and connection between children was also fostered through discussion and collaboration to support affiliation.

Within the context of climate change, promoting social connections in groups could be essential for building individual and community resilience [31]. Satisfying the needs of autonomy and competence in relation to their environmental actions, existential questions, and emotions could be ways of remaining engaged with the issue [59]. Indeed, the satisfaction of basic psychological needs is also strongly linked to one’s motivation to act and pro-environmental behaviors [60]. Wullenkord [59] argued that satisfying basic psychological needs should be a prerequisite for healthy emotional adaptation. When faced with threatening situations, people are more likely to use proactive coping strategies such as action and emotional engagement when their basic psychological needs are satisfied [59]. The self-determination lens was used in the present project to inform the choice of intervention elements to support adaptive coping in children and foster meaning making throughout the workshops.

There is limited research evaluating interventions that address children’s climate change emotions, particularly those that integrate approaches such as creative arts and philosophical inquiry rooted in self-determination theory. Existing research has focused predominantly on climate change education, with minimal exploration of adaptive coping in children. There is a need to document how children respond to discussing climate change emotions in the classroom and to report on their subjective experiences with such interventions.

### Evaluating the social validity of a climate change emotion intervention: study aims and objectives

The present study aimed to document the perceived social validity of a creative arts and philosophy inquiry intervention to address climate change emotions in an elementary school classroom from the perspective of the students and the teacher. The full rationale of the intervention can be found in previous articles published by our research team [46, 61]. The present study aimed to pilot and refine the developed intervention to ensure that it was acceptable and to guide the eventual implementation of an experimental study [62]. Hence, we aimed to document the social validity, which is a concept developed by Wolf [63] to guide the evaluation of new interventions and is broken down into three dimensions. First, the social significance of objectives aims to determine whether the people targeted by the intervention are aware of the intervention’s goals and whether these goals are important to them [63]. Second, the perceived acceptability dimension aims to determine whether intervention procedures are perceived as acceptable by stakeholders [63]. Finally, the perceived effects refer to the importance of the intervention’s results in assessing whether participants are satisfied with the effects of the intervention and whether these effects are consistent with the desired effects [63]. Unexpected, desired, and undesired results of the intervention should also be considered. A secondary aim of the project was to apply self-determination theory to the data to eventually build a theory of change for this program [64]. Qualitative methods, such as semi-structured interviews, are recommended to allow participants to voice their perceptions of the intervention without the constraints of a questionnaire and rating scale, for example [65, 66]. The guiding research questions were *What was the perceived acceptability*,* significance of the objectives*,* and effects of the intervention from the perspective of the students and the teacher? How did the children respond to the intervention? What emotions were expressed by the students? How were the students self-determined during the intervention?*

## Methods

The present study used a descriptive qualitative design to obtain the perspective of students and their teacher. Recruitment was non-probabilistic and consisted of word-of-mouth with elementary schools (levels Kindergarten to Grade 6) with whom the research team had collaborated in previous research studies. One teacher in a public elementary school expressed interest in participating in this project. The sample consisted of 14 fifth-grade students aged between ten and 11 years (7 girls; mean age = 10.29 years) and their teacher. The school was situated in the Laurentians region in the province of Quebec, Canada.

The intervention consisted of weekly workshops that combined creative visual art activities and philosophical inquiry. The breakdown of the intervention can be found in Table 1, with each activity and topic of the discussion. The workshops were led by the first author (TLG), a doctoral candidate in clinical psychology, and two research assistants. All material, including the cameras used for photovoice, was provided by the research team. The photovoice component of the program was partly led by the teacher, who brought the children outside during the week to take the pictures that would then be discussed the following week in the workshop. The pictures were all uploaded to the class computer and projected on the electronic whiteboard. The children were instructed to present their photo in relation to the theme (climate change; the beauty of nature), and the rest of the class was encouraged to react using the prompt questions. The *SHOWED* method [67] was used to foster discussion in photovoice activities, using the following questions: *What do you See here?; What is really Happening here?; How does this relate to Our lives?; Why does this condition Exist?; What can we Do about it?* The presentation of the pictures led to group discussions, and a second related theme (and questions) would be presented to spark the philosophical inquiry. The other sessions of creative activities lasted approximately 20–30 min and were followed by student presentations to share their creation. These presentations led to a group discussion around a related theme. The discussions were performed with the whole class, except on one occasion, where the class was divided in half to allow for a change in group dynamics.

**Table 1:** Weekly breakdown of the intervention

Week	Artistic creation activity	Philosophical inquiry themes and prompt questions
1	Emotion wheel (drawing)	Climate change. What is climate change? What are emotions? How can climate change affect our emotions?
2	Photovoice: The beauty of nature	Beauty. What is beauty? What is nature? How do we find it? How do you feel in nature? Is nature beautiful? Does finding something beautiful mean we feel emotions toward it?
3	Photovoice: Climate change in my environment	Responsibility. How do you react to a picture that is not “beautiful”? Does your picture have as much value as the one from last week? Introduction of the theme of responsibility: what does it mean to be responsible? Who is responsible for the state of the planet? Does everyone have the responsibility to act? Who has the power to change things?
4	The planet in 50 years (drawing)	Change. What is change? Are there different types of changes? How do you feel about change? How has the earth changed in your drawing?
5	Sculpture of something impressive in nature (Lego^®^ or Play-Doh^®^ sculpture)	The strength of nature. What is impressive in the strength of nature? How do you feel about the strength of nature? How do you react when something in nature happens that reminds us of the strength of nature (e.g., forest fires this summer)?
6	Drawing on a rock: something I would like to take care of in nature	Taking care. What does it mean to take care of something? Are there different ways of taking care of things? How can we take care of nature around us? What do we need to be able to take care of nature/others? How can taking care of ourselves give us energy to take care of nature/others? How do you take care of yourself?
7	Poster: my climate change slogan (drawing, collage, painting)	Hope. What is hope? What does it mean to be hopeful? Are you hopeful in the context of climate change? What gives you hope? How can we give other people hope? Can hope increase or decrease on certain days? What influences your levels of hope for the environment? What do you do in moments where you are less hopeful?

### Data collection

The data were collected in four ways: in-class observation, recording of group discussions, student interviews and interviews with the teacher. These various collection methods allowed us to triangulate the data and obtain the perspectives of the parties directly in contact with the intervention to obtain data on the social validity of the intervention.

One research assistant was present during the workshops to take field notes using an observation grid (see Appendix A). This enabled the research team to observe how the intervention was received, what may have happened, different non-verbal signals, and other important elements. These were enhanced by the first author’s (TLG) field notes, which included thoughts, perceptions, and ideas that may have emerged during or after each workshop. Furthermore, reflective notes were also added to these observation notes to list internal biases, assumptions, and beliefs that could be examined to recognize their impact on the results and considered when interpreting the results [68]. Second, each discussion component of the intervention was audio-recorded and transcribed to document the subjects discussed during the workshops and contributed to the exploration of the social validity of the program.

Third, semi-structured individual interviews were conducted with each student who participated in the workshops the week after the end of the intervention. Most children participated in all the workshops; two students had missed one week. These individual interviews were conducted during class time by the first author and two research assistants. Interviews were conducted in quiet environments separate from the class. The research assistants were all trained prior to conducting the interviews. The semi-structured interview guide was developed and piloted in a previous research project led by our team [69] to explore the perceptions of the students of the intervention. The final interview guide can be found in Appendix B. The interviews with each child lasted between 6:58 min and 20:41 min (mean = 11:22 min).

Finally, after the end of the program, an online semi-structured interview was conducted with the teacher to obtain their perceptions of the intervention and its impacts and to document social validity from their point of view. Like the child interview guide, the interview guide for the teacher was adapted from a previous research project (see the final guide in Appendix C). The interview lasted 40:23 min and was audio-recorded using Zoom (Version 5.13.5).

### Data analysis

The qualitative data were analyzed using the software MAXQDA 2022 (Release 22.8.0). All recordings were transcribed and imported into the software for analysis. Thematic analysis with both inductive and deductive approaches was used [70]. After familiarization with the data (transcription and repeated reading; phase 1), the first author (TLG) read through the verbatim and inductively allowed for any code to emerge related to the research question (phase 2). These initial codes were data driven and closely reflected what the participants said. Next, the same data were analyzed deductively, driven by the theory of social validity and its definitions as well as self-determination theory. Hence, the social significance of objectives, the acceptability of procedures and the social importance of effects, as well as the needs for autonomy, competence, and affiliation, were specifically considered within this phase. Once a first coding grid was obtained, a second researcher (JP) applied it to two individual interviews, one focus group session and the teacher interview, to explore any additional codes that might have appeared. Any divergence in coding was discussed between team members. Once the data were initially coded, the analysis was refocused to consider broader themes, using the thematic map generated by the software and discussing it as a team (phase 3). Next, the candidate themes and subthemes were reviewed to consider whether enough data supported each of them, if they were too diverse, or if the pattern within the coded segment was coherent (phase 4). Finally, the themes were named and defined (phase 5).

## Results

Two overarching themes were identified in the data and are described below with the emerging subthemes. The first theme pertained to the acceptability of the intervention. A paradox emerged. The children first felt that they needed to talk about climate change and believed that the workshops were relevant. However, they also wished that they did not have to face this threat or have to think about climate change. This raised questions around the importance of these discussions within the school context. The second overarching theme related to emotions, coping, and the benefits of art. Specific elements related to self-determination theory emerged within this theme. The themes represented how children and the teacher perceived the intervention as being embedded in a larger socio-emotional context.

### Acceptability of the intervention: I want to talk about climate change, but at the same time I wish we didn’t have to

The perceived acceptability of the intervention referred to any elements of evaluation of the intervention expressed by the participants or in the observational data. Overall, the children expressed high general appreciation of the intervention, mentioning that they would recommend it, would do it again, had fun, learned a lot and found it interesting. When asked, all the children said that they would not change anything in the activities and that the instructions were clear. Supporting quotes related to the first theme can be found in Table 2.

**Table 2:** Participant quotes for the theme of the acceptability of the intervention

Theme: Participants’ perceived acceptability of the intervention	
Category	Quote (participant)
General appreciation	Interviewer: So, would you recommend this program to colleagues or other classes? Teacher: Absolutely! Do you need a classroom for this winter? I have a colleague who is waiting for that!
	Well, I really liked it [the intervention in general] and um yeah, I adored it! I would suggest redoing it in another class because it was really cool! (Student 8)
	I really liked it like, I love learning new things like that, and yeah, I really liked doing the activities with you. (Student 6)
	Well, I really liked it [the intervention in general­], it was one of my favorite activities... It's the second favorite activity I ever did in school. (Student 5)
	Well, I really found it cool, and I learned things on the planet and climate change. (Student 14)
Feeling comfortable during the workshops.	I felt comfortable because it was, well, we could really explain well... We could really express ourselves when we were talking. (Student 10)
	Interviewer: Would you say you felt comfortable during the activities? Student 5: yeah. Interviewer: Yeah? What made you feel comfortable? Student 5: Well, that there were already people that I knew.
	Student: Oh yeah, I felt really comfortable! Interviewer: What made you feel comfortable? Student 1: I don’t know, everything was nice, and it wasn't like things I didn't know how to do, and yeah.
Clarity of instructions	Everything was clear, you would explain very well. (Student 10)
	Interviewer: Were there activities that you didn't understand what to do or that the instructions were not clear? Student 7: Um, I don't think so, from what I remember.
	At the beginning I didn't really understand the poster, like I didn’t understand what slogan meant. Like, maybe it would be good to explain it with easier words. (Student 1)
	Well, you know, maybe the for the pictures... Maybe a bit more guidance on like what kind of picture, you know... Maybe more examples… (Teacher)
Time constraints	[I would suggest] Maybe have more activities and maybe more time for the artistic activities. […] I really liked the Lego®, it's just that I did not have the time to do what I really wanted to do. (Student 5) [I would suggest] staying longer, because it went by really fast, like it was how many weeks? Six? Seven? (Student 5)
	Well, I thought it was really interesting that you used art, but we were always missing time to talk about it. (Teacher)
Importance of the intervention and perceived goals	Well, I would have said that the goal was to talk [about climate change] without being alarmist. To talk in an angle that starts with the children, from what they understand, how it makes them feel, their vision of the subject... Because in the end, they are like bombarded with information from everywhere on climate change, you know, school, the TV, newspapers, from this and that, and basically, it is to say, OK, what is THEIR understanding of all this. What is their conception, finally, of what they heard? How they put all that together to get an idea of what it is and what impact it has. Then, ultimately, to talk about it without saying... but without the kind of alarmist tone of we're all going to die anyway. (Teacher)
	Emotions and climate change were the two main themes that were discussed. In the end, it's in line with what we see with our students anyways, but I find that it was easy to make connections. And I liked the discussions because it allowed them, you know, I liked that it always really started from their conceptions, and you let them express themselves. You know, without saying that they are right or wrong. So yeah, I found that really interesting because there aren't many places for that in school. (Teacher)
	Well, [I think the goal was] to see how to, like, change the planet or see how it makes us react or see what people are doing, or us sometimes. (Student 15)
	Uh, maybe it [the goal] was to help you in your study, and maybe it'll help us understand climate change, like maybe there's some people in the class who didn't know what it was. (Student 14)
	Well, I think it [the goal] is more about getting the voice from children, because I think we're the ones who are going to live after all this, not the older people, it's going to be more our generation who are going to have to save the future. So it was to like talk about that... We especially give the voice to adults, but adults, like the older ones, they will not have to endure this... [...] Like, it's not going to happen to them, it's going to happen to us, but they focus more on them. (Student 10)
	Um, [I think the goal was] to make us understand climate change. (Student 8)
	Well, when I knew that the theme would be a bit about climate change, I really liked it. We need to talk about it… and I liked learning new things with you like that. (Student 6)

**Fig. 1:**
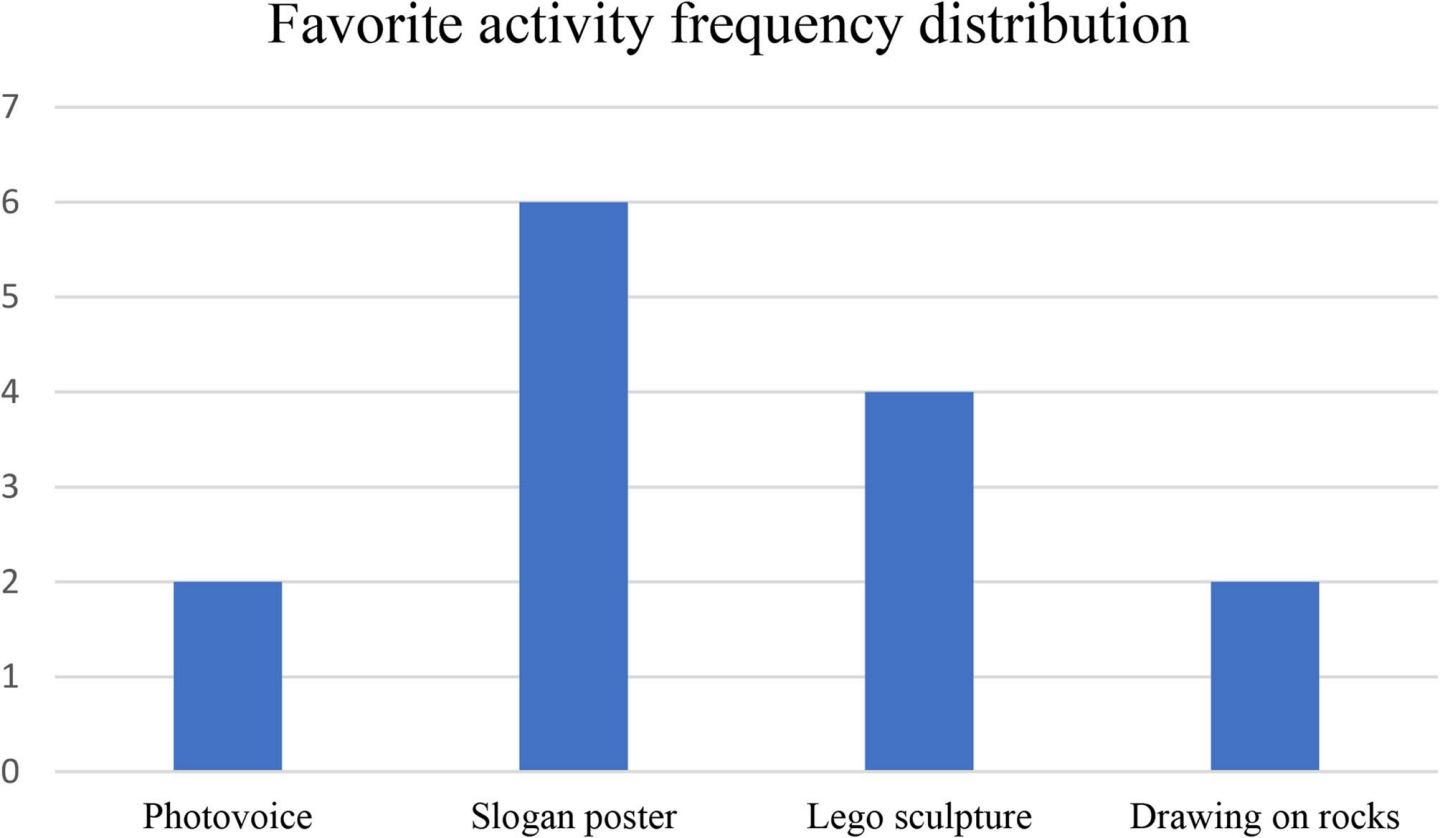
Frequency distribution of students’ favorite activities (*n* = 14)

The students told the interviewers that they felt comfortable during the activities, which was also observed by the researchers and the teacher. For example, many children were excited upon the arrival of the research team, rapidly got into the creation, and gladly shared their artwork with others for all of the workshops. They mostly enjoyed engaging in diverse activities, and all activities stood out for at least one student, as indicated in Fig. 1. The children explained that they enjoyed the photovoice because “we went in the forest/outside.” The school was situated near a park and a small forest area, which is where they went to take pictures of one of the themes. Lego^®^ creation was appreciated because it allowed children to “build with our hands, and just like… touch things.” This sensory dimension was particularly welcomed during class time, where the tasks are often more cognitive. Furthermore, another child described the liberty of creation with Lego^®^, as they could “truly do anything with it.” Hence, including various mediums and activities, seemed to enhance the acceptability of the intervention, as each child could find something that appealed to them.

While the children found the instructions clear and seemed to understand what to do according to the observation notes, the teacher also suggested giving more examples and guiding the children on what to create to allow for more meaningful artwork. Indeed, the teacher reported that many of the children ended up “taking the same photo, so at that level, it was maybe not as interesting, and in the discussions after, there was less substance.” This was consistent with some redundancy noted in group discussions. For example, many children took pictures of cigarettes on the ground to illustrate pollution. Nonetheless, even if children took pictures of the same thing, the discussions that emerged appeared rich and diversified and engaged many children. Thus, the teacher recommended providing better guidelines for taking pictures, as she remained unsure which instructions to give students for the photovoice activities.

No significant distress was observed during the intervention or reported by any of the participants. While many emotions emerged from the discussions and will be elaborated below, none of the students seemed overwhelmed or negatively affected by them. The discussions were calm and respectful; the children listened to one another and built on each other’s responses. Overall, this contributed to acceptability, since the children did not report feeling overwhelmed or significantly upset by any of the workshops, nor was this observed by the teacher or in the researchers’ observation notes.

Many children expressed wanting more time for the creation portion of the workshops. These time constraints were also observed by the teacher, who suggested giving more time for the activities in general, specifically for picture-taking, as well as the discussion component to allow children to share their art creations. The 50 min for the activities were initially chosen because school periods are usually one hour, which could permit any need to transition from and into the workshops and could be easily integrated into the teacher’s schedule. The observational data also indicated that more rigor in time management could have helped ensure that enough time remained to discuss the art creations and engage in philosophical inquiry. While the researchers wanted students to have sufficient time to finish the creative component of the intervention, this often took over half the period, thus leaving less time for discussion and sharing. Nonetheless, the observational notes and group discussion transcripts also highlight that the discussions often seemed to run out of steam within 15–20 min, since fewer children raised their hands, and no further questions emerged.

### Importance of the intervention

A sense of frustration emerged throughout the weeks, as children wished that climate change did not exist and that they did not have to ponder or talk about it. However, the subject of climate change remained important to the students and the teacher, as they had many ideas to share on this topic, and discussions were rich. The perceived goals of the intervention were centered on the pedagogy of climate change. Indeed, the children believed that the goals of the intervention were to educate them on the subject of climate change, discover new things, and just talk about climate change. They believed these goals were reached. Similarly, the teacher believed that the goal of the intervention was to openly talk about climate change without being alarmist. When presented with our goal of discussing climate change emotions, the participants mentioned that this was an important goal but did not bring it up on their own. Although emotions were expressed during the workshops, interestingly, none of the participants identified this as being at the forefront of the intervention. While the teacher believed it was important to create space for climate change emotions, they found it more important to address the pedagogy of climate change:

In my mind, there is a small piece missing, because often, I have the impression, you know, as soon as we start talking [about climate change] it’s like they are stressed, because that’s what is conveyed… So, you know, I guess for me it would be to put that into perspective. […] Students nowadays are more and more anxious, and now, all subjects make them more anxious… and we are talking about it [climate change] and they are stressed. That’s why I would have taken the opportunity to see their conception, and reframe it and yes, give them information so their portrayal of the situation would be more complete. (Teacher)

This perspective highlights the importance of providing teachers with tools to address emotions *and* pedagogy related to climate change with their students. Nonetheless, while the pedagogy around climate change was not directly addressed, many topics and ideas were shared, from the impact of human pollution, such as cars and factories, to the issues around replacing gas cars with electric vehicles and problems with recycling. When some children shared their misconceptions (e.g., that the earth or the sun will explode in the next 50 years), research assistants first let other children respond, before simply adding that science might not support this theory. This type of intervention is aimed at promoting the feeling of a safe space where all perspectives can be welcomed and shared while reframing only if necessary to share science-based information.

Overall, the perceived acceptability of the intervention seemed to be high among students who appreciated their experiences. Both the teacher and the students would recommend the intervention to other classes or would do it again. However, it seemed that the intervention would be more acceptable for the teacher if it integrated pedagogy around climate change. Most importantly, the intervention did not seem to harm the participants from the point of view of the students, of the teacher, or through observation notes.

### Coping with the reality of climate change: sometimes I’m OK, sometimes I’m not, and that’s OK

The second theme that emerged from the data pertained to the emotions experienced during the workshops, the perceived benefits of the intervention, and coping. The supporting quotes for the second theme can be found in Table 3. The recordings of group discussions did not allow the identification of which student was speaking, so the participant number is not included after the quotes.

**Table 3:** Participant quotes for the theme of coping with climate change emotions

Theme: Children’s coping with climate change emotions	
Category	Quote (participant)
Emotions expressed during the workshop	And I feel bad for what I did before… Before I wasn’t really careful. But I realized that it was not good to not respect the Earth (Student, focus group, week 1)
	It makes me angry that people don’t take the time to not put things in the garbage when it goes in the compost… They’re just lazy. (Student, focus group, week 4)
	I’m scared that… You know, if ever something serious happens… (Student, focus group, week 1)
	I’m like afraid for later… (Student, focus group, week 1)
	[I’m not hopeful] Because there are people who are doing well and who stop when the government says to stop, so they stop, but there are people who will continue to pollute the planet when they know they are polluting, and it will always continue. (Student, focus group, week 7)
	[I don’t have hope] because the worst thing about people is that they know what they’re doing it wrong, but they keep on doing it and they know it. But they’re just not being careful. (Student, focus group, week 7)
	I feel fear, anger and sadness, fear because I’m afraid that later it’ll be even worse than this, like 100x worse, that the earth will end up just not being able to cope. I’m angry because everyone, almost everyone does this, they just throw their garbage on the ground and don’t take care of nature. And sad because when I see what’s going on in front of me sometimes, like with pollution, it makes me feel really bad because yeah, I don’t like that. (Student 1)
Perceived effect of the intervention on emotions	Student 5: Before [the workshops], I didn’t see anything, like I thought that everything was normal, but when you think of it, you see the garbage, you see everything. Interviewer: So you feel worse than before? Student 5: Yeah, more angry.
	Now [after the workshops] I feel angrier, because they aren’t doing anything, the government, nobody. Well, except ecologists, that are helping, like my dad who walks a lot. (Student 5)
	Um, well it is the same [as before the workshops], not like sad, but a bit angry at people who do that [pollute]. But there are people trying to help, and then there are others who just throw their garbage on the ground. (Student 4)
	Interviewer: Were there activities that had an impact on your emotions? Student 4: To talk about forest fires. I felt a bit sad… Because I don’t want like… The planet to burn… Interviewer: Would you say that you still feel like that today? Student 4: Yes, well a bit less… Interviewer: What would you say makes you feel less sad today? Student 4: Because we talked about it.
	When I expressed my emotions, I felt like, looser after, I’m not sure how to explain it… (Student 10)
	Well, personally, with my anxiety it really helped me because it was not easy. But, when we went out to see nature, and talked about it, it like reminded me that there are nice things out there… It made me feel good… (Student 10)
	Interviewer: Would you add any emotions to your emotion wheel after all these weeks? Student 10: Umm… disgust, because I feel disgusted that people aren’t being careful.
	Interviewer: Do you think that it [the intervention] had an impact on the way they [your students] feel about climate change? Teacher: Maybe, yes, because it really validated their emotions. You know, sometimes they were stressed, but you said “it’s ok to feel like that”, you know. I feel like that allowed to release a bit of the tension.
	Well, sometimes I just keep my emotions inside… But now we talked about it more and it’s less like… I’m like not going to explode… Well explode in the sense that, you know [gesture that something comes out of their mouth] (Student, focus group week 1)
	Student: Umm, I have less emotions, like when I met you, it was like… I saw that we were talking about climate change it was a bit like… Yeah… And now, it’s not perfect, but there is more calmness Interviewer: More calmness, like you feel calmer inside of you? Student: yes, really.
Affiliation	Yeah, it really made them feel more in relation to one another because it was lots of discussion. And you know sometimes they would build off the idea of others, so yeah, I would say it’s like a community feeling. And especially with this subject [climate change] where they had all the room to express themselves without being told how they should feel or if they are right or wrong… all opinions were valid. (Teacher)
	Yes [I feel more connected to others], like when others speak, well, I understand them, and it’s cool to understand when they are saying. (Student 4)
	Student 3: Well, we [the class] had similar opinions about climate change. Interviewer: Did you feel united in your… Student 3: Yeah, in our action…
	Student 2: I felt a lot of support [from the class], like now I’m less shy to talk in front of the others… Interviewer: Hmm, like in general, or about this specific topic [of climate change]? Student 2: In general.
	Interviewer: What made you feel comfortable during the workshops? Student 5: Well, that there were people I already knew and that we could all talk together.
	I was more on my own [during the activities], I liked to do art in my own bubble. (Student 6)
Competence	Art allowed to get into the subject with something that everyone can do. If you have to draw on rock, you don’t have a choice but to have an idea, and your idea will necessarily represent something you think. (Teacher)
	Well, after we did the drawing, I was more able to like… Well, talk more about it… (Student 15)
	Well actually, like, it made me understand that um, we can make signs and like you know walk in the streets and like tell people to be more careful, and instead of like, well if you see someone throwing a can on the ground, we could just say nothing, but now I will say something because I know. (Student 8)
	Interviewer: Are there activities that made you feel out of your comfort zone? Student 13: Mmm well, maybe when we went to take pictures outside. […] Interviewer: What activity did you prefer doing? Student 13: The pictures!!
	Student 10: Well, basically, expressing myself through how I feel is difficult because I’ll express myself well with people, but not about what I feel… And it’s difficult for me to write it down, to do it. Interviewer: You can say what you think, but not how you feel? Is that what you’re telling me? Student 10: Yeah, that’s it. Interviewer: And the fact that you have to draw it, then put it on paper instead of verbalizing it, how did you find that? Student 10: I found it fun, but *kinda* difficult.
Autonomy	It was good to have fewer examples, but more room for letting things go. (Student 10)
	Student 15: The fact that, like how we did it [in the workshops], when we express ourselves through art, we *kinda* have to be free to decide what to create. Interviewer: You liked that liberty? Student 15: Yeah, really liked it.
	Student 5: Now [that I have more knowledge about climate change], I’m going to be more careful. Interviewer: do you feel more autonomous in your responsibility? Student 5: Yeah, like I can do things too to help…

#### Emotions expressed during workshops

**Fig. 2:**
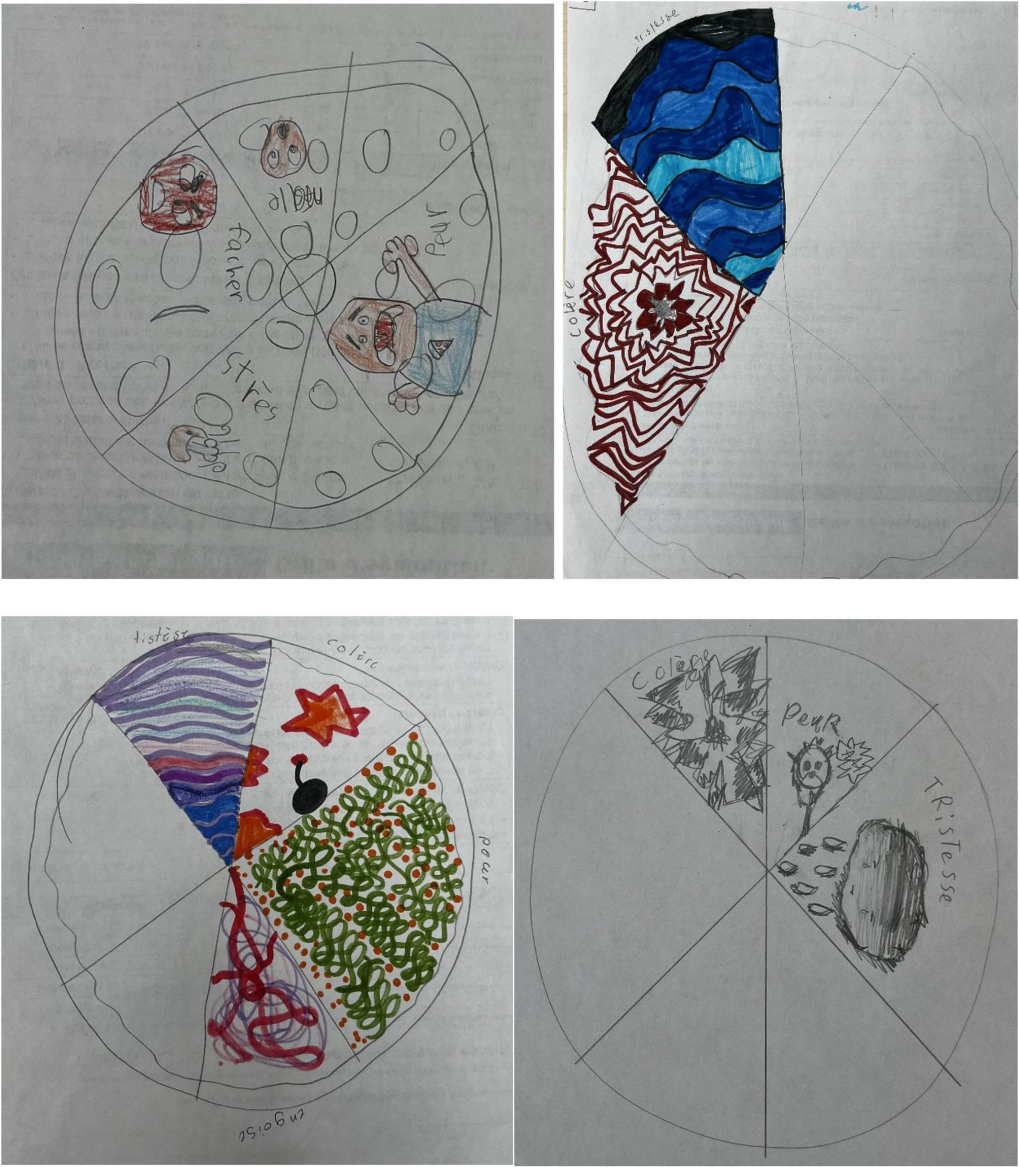
Climate change emotion wheel drawings. Note Translation of emotions written in drawings: Sadness, anger, fear, anxiety, stress

During the weekly discussions about various topics related to climate change, many emotions were expressed. For example, students expressed the anger they experienced when they saw or heard of people and companies that pollute. They felt frustrated that people did not want to “make more of an effort for the planet.” During other discussions, children also expressed feelings of guilt because they themselves also polluted. While sadness was not expressed during the group discussions, it was represented in most children’s drawings of the emotion wheel (Fig. 2). Another emotion that emerged from the discussions was fear. The students expressed being afraid that something bad might happen in the future. It seemed that these emotions were still present after the intervention but that children felt less overwhelmed by them. During the interviews, the children reported that verbalizing their emotions made them feel less intense, as this exchange demonstrated:

- Interviewer: Were there activities that had an impact on your emotions?
- Student 4: To talk about forest fires. I felt a bit sad… Because I don’t want like… The planet to burn….
- Interviewer: Would you say that you still feel like that today?
- Student 4: Yes, well a bit less….
- Interviewer: What would you say makes you feel less sad today?
- Student 4: Because we talked about it.

The topic of hope emerged from some of the group discussions, including hope in humanity, hope in the strength of nature to regenerate, and hope that children will take care of the planet. However, while the final workshop initially intended to discuss the topic of hope by prompting this subject, children wanted to talk about despair. When asked if they felt hope in the context of climate change, the students initially all responded ‘no’. Some felt like there would always be people who polluted no matter what governments did. Some responded that they did not trust governments to push for change, which made them feel despair. Another student expressed that they felt hopeless because people continued to pollute, despite being aware of the environmental impacts of their actions. Students questioned why people acted this way despite knowing the consequences. Interestingly, as quotes illustrate, when children expressed despair, a few also mentioned a hopeful element, balancing both in their discourse and mentioning that it was OK to have both feelings that could coexist even if they could seem contradictory. The discussion also highlighted the importance of acting in the present:

We don’t have a government that’s careful enough, because we’re more concerned about whether something is going to happen, but what we need to do is look at the present, not the future. Because if we go into the future right away, we forget what’s behind us, and that’s what can make things better. Like, stop making promises for the future and focus on what we can do now. (Student 10)

This was also illustrated by children’s perception that electric cars are just a temporary replacement because “electricity also takes a lot of bad stuff […] but also when it’s [the car] no longer good, like when it’s old and doesn’t work, you remove the battery and that’s really not good. [Another student:] Yeah and it’s especially polluting when they’re made.” (Focus group, week 7) Hence, these elements all pointed toward participating children’s need to express their despair in the context of climate change.

#### Perceived effects of the intervention

The students reported that as the intervention unfolded, they experienced a change in the intensity of their emotions. Indeed, as they were provided space to express thoughts and feelings pertaining to climate change, they felt that they were better able to manage their emotional reactions to it. Some children reported feeling that their somewhat distressing thoughts and emotions were still present but not feeling as if they might “explode.” A few mentioned that their sense of anxiety decreased. The students also reported that creating art was a helpful outlet that allowed them to express their emotions. Using various forms of creative arts (e.g., drawing, photography, sculpting) was nonthreatening and made it easier to talk about climate change. However, for other students, their emotions, especially anger, were more common after the intervention. This increase in emotions did not seem to make the students feel overwhelmed or particularly distressed. These children mentioned coping with their emotions by seeking social support from people they trusted (their parents), playing a sport they like, listening to music, or “emptying” themselves of their emotion by expressing it (e.g., crying or screaming in their pillow).

The benefits and theory of change can be explored through the lens of self-determination theory and the satisfaction of the three fundamental psychological needs throughout the data. First, many children reported feeling connected to other students in the class during the intervention because they could belong to the group and feel listened to. Some students even mentioned feeling validated because others felt emotions that were similar to their own. They mostly felt comfortable during the workshops because they were in their classroom with people they knew and trusted. Nonetheless, other students felt that the activities allowed them some time to themselves, especially during art making. The teacher highlighted the openness to ideas and how students built their discourse on what others had said, showing the group cohesion that emerged from the discussions. Hence, the children’s sense of affiliation built during the workshops and seemed to be promoted by being in the classroom with people they knew and trusted, creating a safe space and a sense of belonging in the group, as each idea was heard and validated by others.

Second, creating art helped the children feel competent, as observation notes indicated that it was more fluid for them to express themselves through their artistic creations than to verbally talk about their emotions and thoughts pertaining to climate change afterwards. Notably, the teacher shared that one of their students, who was particularly shy and rarely spoke in class, was able to express themself through their art, often sharing a few words on how they felt about the issue and why they chose a certain subject for their artistic creation. Some students felt that they could better express their opinions and voices on the issue of climate change after the intervention:

Well actually, like, it made me understand that um, we can make signs and like you know walk in the streets and like tell people to be more careful, and instead of like, well if you see someone throwing a can on the ground, we could just say nothing, but now I will say something because I know. (Student 8)

They seemed to have a sense of impact on their environment and confidence that came from learning about the impacts of climate change. Talking about the issue made them feel more competent to act in the future. However, very few children discussed concrete actions, and rather focused on the importance of knowledge before action. Furthermore, the sense of competence also seemed to be fostered through optimally challenging activities that could be adapted to each student. While expressing themselves through arts was initially somewhat intimidating, as the weeks went by, the observation notes reflected that children were increasingly at ease and progressed in the ability to do so. In this vein, children often reported that the most challenging activity, which differed from one student to another, was also their favorite, illustrating that these activities were optimally challenging to foster feelings of competence.

Third, the intervention also appeared to support children’s need for autonomy. With nondirective instructions, the children reported enjoying the liberty of choosing what they could create with their art. This sense of control over their choices was also reflected in the observation notes, whereby the creative arts often involved a wide variety of topics, styles, and messages. Having more knowledge about the impacts of climate change also seemed to increase students’ sense of autonomy in acting. Children reported talking about the issue more with their parents and peers, as well as looking up more information by themselves. As such, both creative arts and open discussions seemed to allow children to feel autonomous in the expression of their emotions and values.

Overall, the perceived effects of the intervention included those of creating a space for expressing many climate change emotions and feeling more in control of these emotions once they could be expressed. These effects could be understood through self-determination theory, as they felt connected with the group, competent to express emotions through arts and share ideas, and autonomously supported.

## Discussion

The aim of this study was to document the perceived goals, acceptability and effects of the intervention from the perspectives of students and teachers. The results support the social validity of the creative arts and philosophical inquiry intervention to promote the expression of climate change emotions. While the children expressed frustration that they had to face the issue of climate change, they believed it was an important topic to discuss in class and appreciated the space given to freely discuss the subject. Climate change may be a difficult subject to integrate into the classroom, as it appears controversial and complex [71]. However, this should not prevent educators from addressing students’ needs of having these safe spaces that allow for emotional reactions [35]. The teacher expressed the need for more tools to support educators who want to teach about climate change. In Quebec, the Ministry of Education intends to reform the science and technology curricula to include current issues such as climate change by 2027 [72]. The Culture and Citizenship in Quebec Program could also guide teachers in talking about climate change using critical dialogues [73]. As such, alongside emotional expression interventions, age-appropriate, scientific-based, and critical pedagogy of climate change should be developed to encourage climate literacy in children [71, 74]. Overall, science modules could integrate the present intervention to allow children to express their emotions as they learn about climate change and its impacts.

Dealing with climate change emotions involves both intellectual and emotional challenges [74], and the creative arts and philosophical inquiry components of the intervention were designed to address both of these aspects. Students generally appreciated the combination of approaches, expressing their excitement and personal preferences for certain artistic mediums and engaging in conversations. The combination of Lego^®^, Play-Doh^®^, drawing, collage, and photovoice allowed each student to be challenged some weeks and more comfortable other weeks, depending on their personal strengths and preferences. Providing choices and various mediums within art making could make the process more meaningful to participants and allow for more engagement, creativity and expression [75]. Furthermore, the diversity in materials and open-ended, unstructured aspects of the intervention were not perceived as being overwhelming by the children in our study but rather supported this need for autonomy in art making. This finding resonates with previous findings whereby art-based interventions are well suited to diverse groups because they meet the participants where they are at [76]. However, the teacher suggested giving more visual examples of what could be created and making things simpler by removing certain choices (e.g., in mediums, of liberty in the topic, etc.) This highlights the balance that is needed between overly guiding students and allowing space for autonomy, which fosters self-determination [56]. Hence, it would be beneficial to further guide teachers within the intervention framework from the beginning and better explain the rationale to them.

The feedback provided by the students and teachers indicated that the intervention aligned with the needs of the children to talk about climate change in schools while creating space for their opinions to be heard by others and their emotions to be expressed. These results are consistent with previous work showing that discussing climate change in the classroom through philosophical inquiry can help children to create meaning and promote hope [50, 77]. Indeed, learning about climate change and its potential impacts can create existential anxiety about the future, as children are confronted with questions about mortality and meaning [47, 78, 79]. Hence, creating meaning has been suggested as an adaptive way of coping with the reality of climate change [9, 53]. Children in our study reported feeling hopeless and threatened by the uncertainty of the future, which is similar to other children and youth populations [6, 13]. This could suggest that they are experiencing a certain level of existential anxiety regarding climate change. Art was a potential way of creating meaning and connecting to their values, as children discussed feeling less overwhelmed by their emotions and wanted to engage in pro-environmental behavior after the intervention had ended. When emotions become overwhelming, people can feel paralyzed, as the threat exceeds their ability to cope with resources [22]. As such, creating spaces for emotional expression and engagement with the issue of climate change can empower children to anchor their action into meaning [9, 13, 80]. As many authors have highlighted, interventions anchored solely in action may risk placing an unrealistic burden on children to ‘solve climate change’ [9, 13, 28, 81]. The present findings seem to suggest that engaging children emotionally can then lead them to *want* to act within their environment, building on their values and motivation. However, results from this study also suggest that children seem to have low agency to act individually and collectively. While their developmental level may render the issue of climate change difficult to understand, this could also be combined with limited exposure to knowledge about the issue. Hence, they may not know what action to do or seek very concrete solutions that are accessible to them, like picking up garbage in the streets. This highlights the importance of offering climate change pedagogy (knowledge) in combination with opportunities for meaningful action as well as emotional expression [9]. Elements relating to compassionate climate change education using various approaches, like climate literature book clubs, outdoor practices, and creative arts could be particularly beneficial [82–84].

The present findings lend support to the benefits of creative arts in providing space for emotional expression [69, 85, 86]. Indeed, the children in this sample expressed many emotions that were voiced by the children and youth in other samples, including guilt, sadness, anger and hopelessness [2, 6, 12, 87]. Some children from our study reported that their negative emotions somewhat increased, as we discussed the topic of climate change. This was especially the case for feelings of anger. Anger is a common climate change emotion in children and adults that can motivate activism and meaningful climate action [88, 89]. Furthermore, adults who experienced anger in relation to climate change were also more likely to have better mental health outcomes than those who were anxious [10]. While few studies exist on children’s experience of anger in the context of climate change, the increase in this emotion observed in this study might be an indicator of future engagement. Other studies reported that learning about climate change triggers the experience of many difficult emotions [90, 91]. A study with older children did not find this association between knowledge and concern (as young people may already be aware of the issue), but nonetheless highlighted the importance of learning coping strategies [74]. Since the topic of climate change can be encountered by children in many ways (family, friends, school, news, YouTube, social media, etc.), it is important to create spaces where emotions can be expressed in a safe and brave environment rather than be avoided [51]. Not only do these spaces allow for emotional validation, but they also provide spaces for holding these emotions and developing tolerance to distress and uncertainty. If an environment is safe, supportive, and nurturing, somewhat overwhelming emotions can be more easily tolerated [92]. Hence, teachers can create nurturing and empowering learning spaces while addressing the issue of climate change with their students using creative arts [35]. When these feelings are not avoided or dismissed, adults can learn to tolerate this discomfort with children, allowing them to move through their climate change emotions [93]. The sensory dimension activated in art making could be one of the pathways by which emotions can be accessed with a step back to learn to tolerate and express them safely [44]. However, a risk also exists in overinterpreting children’s emotions, which can lead educators to make incorrect assumptions about a student’s mental state, inadvertently creating unnecessary psychological narratives around a child’s temporary emotional state [94]. There are many mechanisms of change of art as a form of therapy believed to foster children’s mental health that could explain the present findings. One that is widely accepted is that artistic creation allows children to become more aware of their emotional experience and to express emotions in a non-verbal way, which can encourage emotional regulation [95]. Indeed, creating art permits a visualization of their experience in a safe and less threatening way, overcoming potential avoidance of unpleasant or distressing emotions and leading to acceptance [95, 96]. Finally, art can also lead to positive change by providing a sense of self-determination and control through feelings of accomplishment and authentic self-expression [97].

The initial theme for the last workshop was meant to be ‘*hope’*, to end our visits on a positive note. However, children rapidly shifted the discussion toward despair. The growing body of literature seems to indicate that children are pessimistic toward the future and that this can lead to hopelessness and despair [13, 98]. Despair in the long term is associated with poorer mental health, including more depressive symptoms and suicidal ideation [99]. However, when it is present in a situation that is truly uncertain, such as in the context of climate change, dismissing and avoiding despair can also take a toll on children’s mental health [100]. Indeed, messages of false hope can be perceived as invalidating [93]. Instead, initial evidence suggests that validating these emotions and allowing them to coexist with the feelings of hope that are already present would be a good first step [17]. While adults might feel uncomfortable with these emotions and want to shift the discourse toward a hopeful one, it is instead encouraged to create spaces to explore this despair and ultimately create tools to acknowledge and tolerate this feeling [101]. Nonetheless, it is recommended that children’s experiences of despair be further documented in the context of climate change to better address their specific needs in this regard.

The group context of this intervention study and the satisfaction of the need for affiliation were important elements of the intervention. Indeed, the children from this study expressed the importance of sharing their creations and philosophical thoughts with people they felt comfortable with. This also highlights the need for safe and brave spaces to be well established *before* a group can start addressing difficult emotions and topics such as climate change [35]. Creative arts could prove to be a meaningful way of building supportive environments. The results of this study also highlight the benefits of group approaches in validating experiences of climate change emotions. As a previous scoping review of treatments for eco-anxiety highlighted, having others express a lived experience similar to one’s own can have beneficial, normalizing, therapeutic effects and reduce feelings of solitude [31]. Moreover, children could also make sense of others’ experiences through their art, creating another avenue for proximity and empathy [102]. This also emphasizes the importance of spreading the intervention over a certain number of weeks to allow the feeling of affiliation to be developed [103]. In essence, discussions within the context of the school classroom, similar to group therapy, could have allowed the children to develop new understandings of their own experience through others’ reflections and support [104].

Finally, children in our sample recommended giving more time for creations, without suggesting removing time for the introduction and discussion components. This resonates with previous research, in which children expressed a preference for having one main activity per session (i.e., either art making or engaging in a philosophical inquiry), giving them more time for creation [103]. Interestingly, the teacher from this study recommended allotting more time to group discussions and less time for creation. This could reflect the children’s preferences to perform activities considered fun, whereas the teacher might want to prioritize learning experiences. The amount of time spent on each component of the intervention should be adequately balanced to address the needs of both the students and the teachers. A sense of frustration could emerge if children systematically do not have enough time to finish their creations, and they might not gain the benefits of sharing these creative arts and discussing philosophical issues in class if there is not enough time remaining to do so. As such, one recommendation would be to separate the activities into two periods within the same week to first take part in the artistic creation and later share these creations with the rest of the class and initiate a philosophical inquiry. This could allow for more flexibility in the time spent on creation and discussion.

### Strengths and limitations

The present study addresses the critical and timely issue of children’s emotional responses to climate change. The intervention builds on the strengths of the complementary approaches of creative arts and philosophical inquiry, which are both easily accessible to children and schools. The method used to document the acceptability of the intervention explored the subjective experience of participants by including their voices through qualitative components. Furthermore, the results were triangulated via multiple data collection methods. By using both inductive and deductive methods of thematic analysis, it was possible to capture participants’ perspectives while applying relevant theories to the data. This also allowed for both positive and negative outcomes to arise. To reduce the potential interviewer effect, three research assistants led the interviews along with the principal investigator, and they paid particular attention to their biases and how these biases could impact their posture during the interviews. Furthermore, potential coding biases were safeguarded by having multiple coders, discussing results between researchers, and holding a diary to explore potential biases.

However, the school-specific context and lack of access to parents’ perspectives limit information on participants’ characteristics and the generalizability of the results to other cultural or educational contexts. The sample was recruited non-probabilistically and was quite small, which can also impact the generalizability of the results. This study should be conducted with more diverse samples, in different schools or social contexts, and with children of different ages to better understand who might benefit most from the intervention. There is also the possibility of desirability bias in the participants’ responses, as children might have wanted to give the ‘right answer’ or be perceived in a positive light. While children were told that all feedback was welcomed and that specific questions asking about what could be changed if the same intervention was done again, the children may have had the impression that they had to give positive feedback about the intervention. This bias was potentially reduced by reiterating that any information could be shared and that their personal experiences and perspectives (i.e., that there were no possible wrong answers) were sought. Yet it is still possible that this bias influenced the study’s results. There is also a limitation in the length of the intervention that is quite short for a school-based intervention.

## Conclusions

The results of this study indicate that the seven-week creative arts and philosophical inquiry intervention was acceptable for a class of elementary school children and their teachers. The participants believed that it was important to talk about climate change in the classroom. The children expressed many emotions through their art, which facilitated the discussion of many difficult existential questions. This included the topic of despair, whereby children mentioned that they did not feel hope in the context of climate change. These results can inform researchers and educators of the importance of creating spaces to express these difficult emotions without invalidating them. Ultimately, children and teachers indicated that the topic of climate change should be discussed in class and that creating space for emotions that can arise during these discussions is important. Creative arts allow children to express these emotions non-verbally, whereas philosophical inquiry allows them to explore different questions they might have on the topic. Although some children did voice some negative emotions linked to climate change, no negative impacts were highlighted by the participants. Overall, these results suggest that the intervention was acceptable from the perspective of the children and their teacher, indicating that a larger scale study to evaluate the impacts with a larger sample would be appropriate [62]. Our findings, while preliminary, suggest that creative arts and philosophical inquiry could be integrated into elementary school classrooms to foster meaning making as children learn about climate change and its consequences.

## Supplementary Information

Supplementary Material 1.

## Data Availability

The datasets generated and/or analyzed during the current study are not publicly available due to the nature of qualitative data that might contain identifying information through the interview (e.g., speech slurs) but are available from the corresponding author upon reasonable request. The present manuscript contains excerpts of the verbatim content that support each of the themes presented. Full data are not made available to ensure the confidentiality of the participants.
