# Supplementary material for: I want to talk about climate change, but I wish I didn’t have to: A descriptive qualitative study of an intervention combining creative arts and philosophical inquiry to help elementary school students cope with climate change emotions
Source: BMC Psychol. 2025 Dec 8;13:1339. doi: 10.1186/s40359-025-03589-w (PMC12683917; doi:10.1186/s40359-025-03589-w)
Supplement: Supplementary file 1 — Supplementary Material 1. [file 40359_2025_3589_MOESM1_ESM.pdf]

APPENDIX A – Observation Grid used by researchers during the in-class intervention [French]

Observation guide			
Date :	Activité :	Début :	Fin :
**Domaine d'observation**	**Observations**		
Quelles sont les réactions des élèves à cette activité ?			
Quelles sont les attitudes des élèves à l'égard de l'activité ?			
Quels sont les événements qui se sont produits durant l'activité ou les événements notables qui l'ont influencée ? (Par exemple, interruptions, célébrations, etc.)			
Quels sont les différents rôles que les enfants adoptent durant l'activité ?			
Quelles sont les interactions entre les personnes ? (Par exemple, la nature des interactions, la raison pour laquelle elles se produisent, les émotions exprimées) ?			

Quelles sont les relations entre les élèves pendant l'activité ?	
Que disent et font les gens ?	
Quels sont les messages non verbaux ?	
Quelle est l'atmosphère générale ?	
Comment l'environnement est-il structuré ? (Par exemple, cadre physique, disposition des sièges)	

APPENDIX B – Children semi-structured interview guide [French]

Entrevues avec les enfants – Guide de questions semi-structuré

1. Introduction

- Description du projet recherche
  - *Comme tu le sais, on est venus animer des séances d'art et de photo avec des discussions dans ta classe. On se demande comment les enfants ont aimé ça, s'il y a des choses à améliorer, comment le tout s'est passé pour toi.*
- Description du déroulement et de leur participation
  - Quelques précisions :
    - *Tu n'es pas obligé de répondre à des questions qui ne te tentent pas*
    - *Tu peux prendre le temps de penser avant de répondre*
    - *Dis-le-moi si tu ne comprends pas une question*
    - *Tu peux utiliser les mots que tu veux pour bien représenter ce que tu penses et pas ce que tu penses que je veux entendre ; il n'y a pas de mauvaise réponse, ce qui est important pour moi est de bien comprendre ce que tu veux me partager*
- Consentement
  - Débuter l'enregistrement
  - *Je vais enregistrer notre discussion pour pouvoir me rappeler ce dont nous avons parlé. Je vais transcrire tout ce qu'on a dit et enlever ton nom, puis je vais supprimer l'enregistrement. Ensuite, je vais comparer ce que tous les élèves ont dit pour trouver des ressemblances et des différences dans ce qui est dit pour améliorer notre projet. À aucun moment on pourra identifier qui a dit quoi, donc ta participation reste confidentielle. Es-tu d'accord pour que j'enregistre et pour participer à cette discussion? [Sinon, s'il ne peut pas faire l'entrevue, remercier l'enfant et ne pas faire l'entretien.]*
- *Partir l'enregistrement, dire le nom du/de la participant(e) ou leur demander de le dire.*

Guide entretien validité sociale intervention

Nous avons fait plusieurs ateliers ensemble dans les dernières semaines, peux-tu me nommer ce que nous avons fait? [Rappel des ateliers]

- Peux-tu commencer par me parler de ton **expérience générale** avec les ateliers? Comment est-ce que tu as trouvé ça?
- Quels ont été tes **défis** durant les ateliers?
  - Quelle a été l'activité que tu as trouvé la plus difficile (*qui t'a sortie de ta zone de confort*)?
- Quelles activités as-tu **préféré** faire au cours des dernières semaines?
  - Qu'est-ce qui a fait en sorte que tu préfères cette activité?
- Quelles activités as-tu le **moins aimé**?
  - Qu'est-ce qui a fait en sorte que tu aimes moins cette activité?

- Si je te disais que je veux revenir faire des activités d’art dans une autre classe, qu’est-ce que tu me **conseillerais** pour que ce soit plus plaisant pour les élèves?
    - Qu’est-ce que tu changerais dans la façon de faire les activités?
- Est-ce qu’il y a des activités que tu n’as **pas comprises** ou pour lesquelles les **consignes** n’étaient pas claires?
    - Qu’est-ce qui aurait aidé?
- Est-ce que tu te sentais **à l’aise** durant les activités?
    - [*Non*] Qu’est-ce qui aurait pu te faire sentir plus à l’aise?
    - [*Oui*] Qu’est-ce qui a fait que tu te sentais à l’aise?
- Qu’est-ce que tu penses était le **but** de venir faire ces activités?
    - [*Parlent d’un but*] Dirais-tu que nous avons atteint ce but? [Comment?]
    - [*Ne sait pas*] En fait, le but était de trouver du temps pour parler des changements climatiques et des émotions que ça pourrait amener, dirais-tu que ça a été le cas?
    - Selon toi, qu’est-ce qui aiderait le mieux à atteindre ce but?
- Est-ce que tu as remarqué **des changements** de faire les activités d’art et les discussions de groupe :
    - Dans ta façon de te **sentir**? *Oui -> comment est-ce que ça a changé? Non -> qu’est-ce qui aurait aidé?*
    - Dans ta façon de **penser**? *Oui -> comment est-ce que ça a changé? Non -> qu’est-ce qui aurait aidé?*
    - À **l’école**? *Oui -> as-tu des exemples? Non -> qu’est-ce qui aurait aidé?*
    - À la **maison**? *Oui -> as-tu des exemples? Non -> qu’est-ce qui aurait aidé?*
- Dans quelles situations penses-tu que les activités qu’on a faites pourraient **aider** les autres enfants du primaire?
    - Pourrais-tu m’en dire plus sur comment ça pourrait les aider?
- Qu’est-ce qui a été le plus **aidant pour toi** dans les activités d’art/photo?
    - Pourrais-tu m’en dire plus sur comment ça a été aidant pour [x].
- Qu’est-ce que tu as pensé des discussions qu’on a eues en classe sur les différents thèmes liés aux changements climatiques?
    - Quels thèmes de discussions liés aux changements climatiques que nous n’avons <u>pas</u> abordés est-ce que tu penses seraient bien à discuter en classe?

- Est-ce que de faire les activités a eu un **impact** sur ta **perception** des (*comment tu comprends/ comment tu penses aux*) changements climatiques?
    - Si oui : Pourquoi est-ce que tu penses qu'il y a eu ces changements après les activités d'art?
- Est-ce que de faire les activités ont eu un **impact** sur tes **émotions** par rapport aux changements climatiques?
    - Si oui, lesquelles?
- Comment est-ce que de participer à ces activités pourrait changer le sentiment **d'autonomie** des élèves (*se sentir à l'origine de ses actions, choisir ce que tu fais*)?
    - Dirais-tu que ça a été le cas avec toi?
- Comment est-ce que de participer à ces activités pourrait changer le sentiment de **compétence** (*se sentir capable faire des choses de différents niveaux de difficulté*)?
    - Dirais-tu que ça a été le cas avec toi?
- Comment est-ce que de participer à ces activités pourrait changer le sentiment de **connexion** aux autres (*se sentir connecté et supporté par d'autres personnes*)?
    - Dirais-tu que ça a été le cas avec toi?
- Penses-tu que ces activités ont influencé la façon dont tu t'exprimes et partage tes pensées/idées?
    - Oui -> comment?
    - Non -> qu'est-ce qui aurait aidé?

Conclusion

- As-tu autre chose à ajouter par rapport aux activités?
- As-tu des questions pour nous?
- [Remercier de leur participation tout au long]

APPENDIX C – Teacher semi-structured interview guide [French]

Guide d'entrevue semi-dirigées avec les enseignants.es

Bien que les questions soient présentées dans un ordre distinct, elles pourraient être posées dans un autre ordre durant l'entretien pour permettre aux participants.tes de partager leur expérience librement.

1. Introduction

- [Réitérer le consentement]
- L'objectif de ces entretiens est de mieux comprendre votre perception de l'intervention et des impacts sur vos élèves.
- Il n'y a donc pas de bonne réponse, on cherche à mieux comprendre les bons coups et les moins bons coups selon votre perspective.

2. Perception des ateliers

- Comment décririez-vous le niveau d'engagement des enfants dans les ateliers?
- Est-ce que vous avez des commentaires sur la façon dont les activités se sont déroulées?
- Quels ateliers étaient plus appréciés par vous?
    - Par les élèves?
- Quelles sont vos recommandations pour le programme, s'il était à refaire?
    - Qu'est-ce qui serait à refaire et à changer selon vous?
- Avez-vous des commentaires sur les procédures et la faisabilité du programme?
    - Est-ce que les ressources matérielles étaient adéquates?
    - Est-ce que le contexte se prêtait bien aux ateliers (la classe, le temps, la disponibilité des enfants, votre disponibilité, le nombre d'élèves, etc.)?
    - Qu'est-ce que vous avez trouvé du processus des caméras (nombre de caméras, aller dans du temps de classe, etc.)
- Consignes
    - Comment avez-vous trouvé les consignes des ateliers?
    - Est-ce que le nombre de participants, le temps et les ressources permettaient une participation significative? Comment est-ce que l'on aurait pu améliorer ceci?
- Est-ce que vous recommanderiez ce programme à des collègues dans d'autres classes?
- Qu'est-ce que vous avez spécifiquement aimé du programme?
    - Moins aimé?

3. Santé mentale des enfants

- Que pensez-vous était le but du programme?
    - Pensez-vous que nous avons atteint ce but?
    - Est-ce que vous trouvez que c'est un but important?
    - Pourquoi pensez-vous que c'est important de parler des émotions par rapport aux changements climatiques dans le contexte scolaire?

- Pensez-vous qu’il pourrait y avoir des changements sur comment les élèves perçoivent les changements climatiques après avoir fait les ateliers?
- Comment pensez-vous que les ateliers ont eu un impact sur la façon dont les jeunes se sentent par rapport aux changements climatiques?
- Trouvez-vous que les activités ont permis l’expression des émotions?
    - Hors des ateliers?
- Selon vous, comment est-ce que l’art peut aider les enfants à se sentir plus :
    a) Compétents
    b) En relation avec les autres
    c) Autonomes
    - Est-ce que vous pensez vous pensez que les activités que l’on a faites ont permis cela?
- Diriez-vous que ce genre d’atelier serait un bon ajout lorsque les enfants apprennent à propos des changements climatiques?
    - Comment serait-ce aidant? Ou comment est-ce que ça pourrait être plus aidant?
- Avez-vous remarqué d’autres impacts sur la santé mentale de vos élèves suite aux ateliers, qu’ils soient positifs ou négatifs?

4. Remercîments

- Nous vous remercions de nous avoir accueillis dans votre classe et pour votre participation précieuse.
